# National hospital mortality surveillance system: a descriptive analysis

**DOI:** 10.1136/bmjqs-2018-008364

**Published:** 2018-10-08

**Authors:** Elizabeth Cecil, Samantha Wilkinson, Alex Bottle, Aneez Esmail, Charles Vincent, Paul P Aylin

**Affiliations:** 1 Primary Care and Public Health, Imperial College London, London, UK; 2 Department of Non-communicable Disease Epidemiology, London School of Hygiene and Tropical Medicine, London, UK; 3 Health Services Research and Primary Care, University of Manchester, Manchester, UK; 4 Experimental Psychology, University of Oxford, Oxford, UK

**Keywords:** hospital mortality, quality of care

## Abstract

**Objective:**

To provide a description of the Imperial College Mortality Surveillance System and subsequent investigations by the Care Quality Commission (CQC) in National Health Service (NHS) hospitals receiving mortality alerts.

**Background:**

The mortality surveillance system has generated monthly mortality alerts since 2007, on 122 individual diagnosis and surgical procedure groups, using routinely collected hospital administrative data for all English acute NHS hospital trusts. The CQC, the English national regulator, is notified of each alert. This study describes the findings of CQC investigations of alerting trusts.

**Methods:**

We carried out (1) a descriptive analysis of alerts (2007–2016) and (2) an audit of CQC investigations in a subset of alerts (2011–2013).

**Results:**

Between April 2007 and October 2016, 860 alerts were generated and 76% (654 alerts) were sent to trusts. Alert volumes varied over time (range: 40–101). Septicaemia (except in labour) was the most commonly alerting group (11.5% alerts sent). We reviewed CQC communications in a subset of 204 alerts from 96 trusts. The CQC investigated 75% (154/204) of alerts. In 90% of these pursued alerts, trusts returned evidence of local case note reviews (140/154). These reviews found areas of care that could be improved in 69% (106/154) of alerts. In 25% (38/154) trusts considered that identified failings in care could have impacted on patient outcomes. The CQC investigations resulted in full trust action plans in 77% (118/154) of all pursued alerts.

**Conclusion:**

The mortality surveillance system has generated a large number of alerts since 2007. Quality of care problems were found in 69% of alerts with CQC investigations, and one in four trusts reported that failings in care may have an impact on patient outcomes. Identifying whether mortality alerts are the most efficient means to highlight areas of substandard care will require further investigation.

## Introduction

There is greater emphasis on accountability and performance monitoring in healthcare, especially in the wake of a number of high-profile health service scandals in the UK.^1–3^ In 2007 the Dr Foster Unit at Imperial College began generating monthly mortality alerts for specific diagnosis and procedure groups for National Health Service (NHS) hospital trusts (all English acute non-specialist trusts). The Imperial mortality surveillance system was critical in detecting problems at Mid Staffordshire NHS Foundation Trust. The resulting Public Inquiry recommended that all hospital trusts should have systems providing real-time information on mortality, patient safety and quality of care.^3^

Monitoring mortality is now an integral part of healthcare. Many healthcare systems now use routine administrative data to calculate quality indicators, including hospital mortality. Examples of these are the analytic toolkits, provided by commercial companies such as Dr Foster (UK), Telstra (Australia) and 3M (USA). However, to our knowledge, there is no published literature on actions taken in response to these toolkits. Case note reviews (or databases) have been used for some years to monitor outcomes such as mortality in various settings^4^ for specific conditions or surgical procedures.^5 6^

The strength of the Imperial College system is its coverage of all acute English NHS public hospitals at the diagnosis and procedure group level for patients admitted for any reason. As a result, the alerts may highlight possible failings that can be more easily isolated and investigated by hospital trusts than summary measures of mortality. The basis for these mortality alerts in the Imperial College Mortality Surveillance System is a statistical process control chart^7^ using national hospital administrative data, Hospital Episodes Statistics (HES).^8^ The aim is to alert trusts when a sustained higher than expected mortality rate exceeds a threshold set at a 0.1% probability of a statistical false alarm in the preceding 12 months.^9 10^ Each alert is reviewed individually to decide whether to notify the trust; alerts are based on recent activity within the trust of a specific condition and therefore may be easier to investigate than summary (overall) measures of mortality such as the hospital standardised mortality ratio. Alerts are not sent to trusts if (1) they represent small numbers of deaths (expected <5) or (2) they are repeat signals for which the trust has already been alerted to within the previous 9 months. Alerts for some procedure and diagnostic groups have been withheld in response to hospitals raising concerns about the reliability of the coding (eg, percutaneous transluminal coronary angioplasty). Imperial College notifies the chief executive of the alerting trust by letter. An example letter is shown in online supplementary file 1. The content details the rationale for sending the letter and presents the statistical process control chart and statistics for the preceding 12 months (number of admissions, deaths, expected deaths, relative risk, c-statistic and the probability of the alert occurring by chance). Imperial College sends copies of all the alerts (including those not sent to trusts) to the Care Quality Commission (CQC). The CQC’s purpose is to monitor, inspect and regulate services within the NHS trusts and to encourage services to improve. It uses the Imperial College Mortality Surveillance System as part of its ongoing monitoring of trusts’ performance. The CQC may write to a trust to request an investigation and response.^11^

10.1136/bmjqs-2018-008364.supp1 Supplementary data

The mortality surveillance system’s overall purpose is to support hospitals in identifying potential quality issues in the care they provide. However, this process demands significant resources from the CQC, Imperial College and the alerting trusts. Uncertainty still exists about the sensitivity of systems that use routinely collected hospital data and the way in which healthcare providers respond to alerts.^11^ We aimed to carry out a descriptive analysis of alerts generated by the Imperial College Unit since 2007 (to 2016), and present the outcome of investigations elicited by the CQC for a subset of alerts (sent between 2011 and 2013) and to assess the impact of the alerts on the hospitals concerned.

## Methods

### Mortality alerts

The Imperial College Mortality Surveillance System generates monthly alerts which are specific to a procedure or a diagnosis group. Diagnosis groups are based on the Agency for Healthcare Research and Quality’s Clinical Classification Software (CCS)^12^; a list of the conditions covered by the system is provided in online supplementary file 2. The Dr Foster Unit at Imperial holds records of all alerts generated and sent to trusts. We created a database of the alert information using data extracted from the alert letters. We assembled a further database of trusts mergers and closures using data supplied by the CQC. We carried out a descriptive analysis of all alerts generated from April 2007 to October 2016. We investigated alerts by diagnosis or procedure group, by year, and for the top 5 alerting trusts. We investigated trends using a non-parametric test for trend across ordered groups.^13^

10.1136/bmjqs-2018-008364.supp2 Supplementary data

### Data collection at CQC

**Figure 1:**
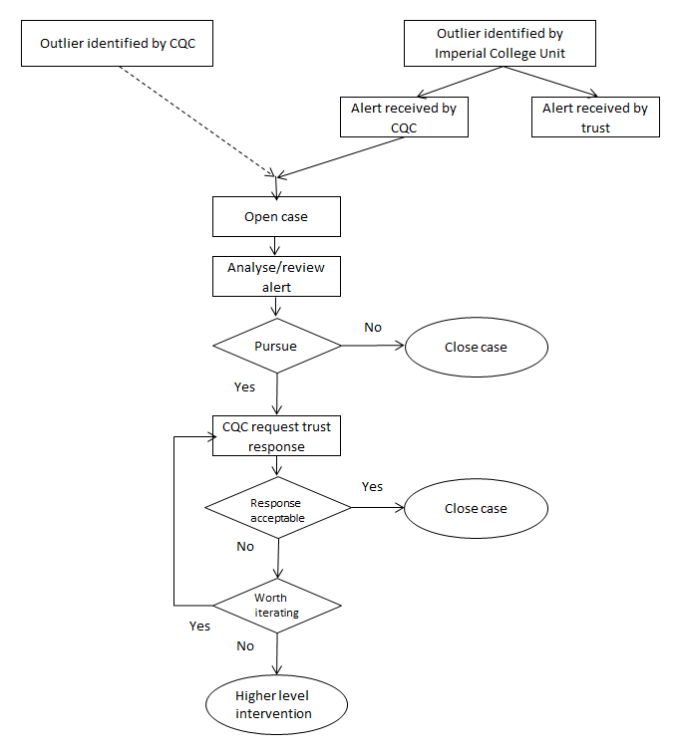
Process chart outlining the Care Quality Commission’s (CQC) follow-up of mortality alerts.

Copies of all the alerts, including those not sent to trusts, are sent to the CQC. The CQC has been running its own mortality surveillance system using the HES.^11^ On the basis of either system, the CQC can open a case by writing to a trust to request a trust-level investigation and response.^11^ When the CQC receives a response from the trust, a panel of clinicians and analysts reviews the information. At this stage the CQC may close the case (and request follow-up by their regional teams) or request further information. The process chart outlining the CQC’s follow-up of mortality alerts is shown in figure 1.

We systematically reviewed the documents held by the CQC for each mortality alert generated by Imperial College, focusing on a subset of alerts sent between 2011 and 2013. We chose this period to allow sufficient time for investigations to be completed, and to limit the scope of the study to ensure data collection was achievable with our resources. One reviewer (SW) collected and compiled the CQC data at the CQC site to protect the potentially sensitive information contained in the documents. We checked the quality of the data collected at random to ensure accuracy; for example, dates were checked to identify non-sequential correspondence. We only had access to documentation relating to alerts triggered by Imperial College Unit and did not have access to information relating to alerts generated by other monitoring systems.

The CQC checked the data that we collected prior to releasing the information to us. We developed a data collection form in Excel, which we piloted and refined. In the pilot phase the data collection comprised up to 121 pieces of data per alert; however, these were progressively refined to a core of 44 relevant pieces of information, collected from 4 document types. The documents described in table 1 were the main source of information in our study. As letter dates were predominantly sequential, that is, trust responses should follow the CQC data requests, we checked dates regularly to identify non-sequential letters.

**Table 1:** Summary of data and documents available at the Care Quality Commission (CQC) relating to mortality alert reviews

Document name	Document description	Key data items
Analyst report	Created by CQC analysts, collates CQC information for the trust and recent mortality data.	Decision to pursue or close, and date compiled.
CQC information request	Letters sent to trusts from the CQC requesting information.	Letter dates.
Trust response	Letters received by the CQC from trusts, containing responses to information request.	Details of case note reviews and letter dates.
CQC assessment of response	Summary document used by the CQC panel that summarises the case and the information received from the trust.	Results of case note reviews, action plan status, panel recommendations and dates.

Where possible, we used the record of the CQC panel meeting, the ‘CQC assessment of response’, as the source of summary information. We used the findings from trust investigations, as summarised by the CQC analysts, to classify the trust’s conclusion as to the reasons for the alert. Where the CQC analysts noted that trusts had identified deficiencies, we included these findings in our data collection. The CQC analysts responded in five broad ways: (1) no evidence of underlying problems in care; (2) mortality alert was due to an unusual casemix; (3) mortality alert due to inaccuracies in coding; (4) problems in care were evident; and (5) a combination of care and coding problems. We therefore classified their response as (1) no improvement required; (2) casemix; (3) coding; (4) care; and (5) care and coding. Where the CQC analysts had not summarised the findings from a trust, the reviewer classified the trust response using information taken directly from ‘Trust response’ documents. For example, if the trust referred to plans to improve both care and coding, then we classified the alert as ‘care and coding’. We analysed the recorded frequency of trusts reporting that patient outcomes were likely to have been affected by issues identified. No sensitive information was stored in the final data set.

We reported trust findings across all diagnosis/procedure groups and also focused on *septicaemia (except in labour)* alerts, a common reason for an alert, which, for simplicity, we term sepsis alerts. We defined a multiple alert as two or more alerts occurring within a trust for the same condition within 12 months of each other. We combined these multiple alerts into a single event to determine whether multiple alerts were more likely to signal problems with quality of care than single alerts. We tested difference in proportions between groups using the χ^2^ test.

### Patient involvement

This paper is part of a larger project evaluating a national surveillance system for mortality alerts. There were two patient representative members of the Scientific Advisory Group who contributed to the development of the research question and outcomes of this study. There was a consultation with members of the public through peopleinresearch.org, and five participants attended a focus group which discussed mortality alerts and the justification for using personal data to generate them.

## Results

### All alerts

Between April 2007 and October 2016, Imperial College generated 860 alerts (all alerts were sent to the CQC for reference). Of these 317 alerts (37%) were multiple alerts (alerts for the same trust and condition within 12 months of each other). Of the alerts, 76% (654/860) were sent to 143 trusts and the Department of Health; 206 were withheld. The mean number of alerts sent each year was 68, with another 22 withheld. There was no statistically significant evidence that the proportion of alerts sent to trusts had changed over the course of the study. Annual volumes of alerts sent have varied over time, ranging from 40 to 101 alerts (table 2). There also did not appear to be any seasonal trends in the number of alerts sent; the average number of alerts sent each month was 7. The time between cumulative mortality rates exceeding a set threshold (generating an alert) and the letter sent to the alerting trust ranged from 13 to 22 weeks, with a median of 15 weeks.

**Table 2:** Description of sent alerts

Characteristics	Alert count (%), n=654
Year	
2007*	46 (7.0)
2008	76 (11.6)
2009	101 (15.4)
2010	60 (9.2)
2011	85 (13.0)
2012	65 (9.9)
2013	57 (8.7)
2014	40 (6.1)
2015	70 (10.7)
2016*	54 (8.3)
Diagnosis or procedure	
Diagnosis	514 (78.6)
Procedure	140 (21.4)
Top 5 diagnoses	
Septicaemia (except in labour)	75 (11.5)
Coronary atherosclerosis and other heart disease	32 (4.9)
Urinary tract infections	28 (4.3)
Fluid and electrolyte disorders	26 (4.0)
Acute myocardial infarction	25 (3.8)
Top procedure	
Coronary artery bypass graft (other)	26 (4.0)
Top 5 most alerting trusts	
A	20 (3.1)
B	15 (2.3)
C	13 (2.0)
D	13 (2.0)
E	12 (1.8)

Number of alerts sent 2007–2016, showing top alerting diagnoses and procedures, years and anonymised top alerting trusts.

*Incomplete years.

Sepsis was the most commonly alerting CCS group with 96 alerts (75 sent), which accounted for 11.5% of all alerts sent (table 2). *Coronary artery bypass graft (CABG) (other)* was the most commonly alerting procedure (4% of all alerts); ‘Other’ in this case means not first-time, isolated CABG.

Twenty out of 154 acute NHS trusts (13%) had never received an alert by 2016. Of the 143 notified alerting trusts over the study period, 20 (14%) received a single alert while 123 (86%) received more than one alert. Seventy-one (50%) trusts received more than one alert for a single condition over the study period, while 43 (30%) trusts alerted more than once for a single condition within 12 months.

Sepsis had the highest proportion of multiple alerts. The highest number of alerts sent to a single trust, for a single condition, was six alerts for *CABG (other).*

### CQC investigations

We examined a subset of CQC records for 204 alerts sent to 96 hospital trusts by Imperial College for alerts occurring between 2011 and November 2013.

Alerts are first reviewed by the CQC analysts who compile a report using CQC data on the trust. On average, the time between an alert letter and the analyst’s report was 6 weeks (median). At this stage, one in four (50/204) alerts were not pursued by the CQC, and these cases were closed. In 154 alerts, the CQC asked the trust to investigate the alert.

The information on why the CQC decided to pursue or not was limited. In 62% (127/204) of all cases, there was no information found on this decision. Of those not pursued the most common reason (eight cases) was because there was an ‘overlap with other alert’. These could be due to a CQC triggered warning or a multiple Imperial College alert. There was only a single case where the reason for not pursuing was recorded as a ‘repeat alert’. However, the CQC seemed less likely to pursue a case when the trust had previously alerted for the same condition within 12 months, compared with those that had not, 44% (11/25) vs 79% (143/179) (p=0.001).

On receipt of a trust response, a panel of clinicians and analysts reviews the information received. At this stage the CQC may close the case (and request follow-up by their regional teams) or request further information. On average, cases were open with the CQC for 23 weeks (median). This is the number of weeks between the date on the mortality alert letter and the end of the CQC investigation. The longest case that we reviewed was 59 weeks, and the shortest case was pursued for 9 weeks. In total, 3856 sets of patient notes were reviewed as a result of the mortality surveillance system between 2011 and 2013.

In 90% (140/154) of investigated cases, trusts reported results of a case note review to the CQC. On average these reports included details of 24 patients (median) per alert (range 5–100). Case note reviews focused mostly (85%, 119/140) on the period when the alert was generated, although information on periods covered in the audit was missing in 12 of these cases and audits covered periods that were not during the alert period in 9 cases. The median time for investigations involving case note reviews was 1 year (364 days) and ranged from 30 to 546 days. Investigations at the trusts were not standardised and carried out according to local practice.

In their local investigations, hospital trusts found areas of care that could be improved for 69% (106/154) of the cases pursued by the CQC. Examples of these areas of care highlighted by trust investigations are shown in box 1.

Box 1 **Examples of areas of care that could be improved or other areas that could impact on care, highlighted after trust investigations**

**Examples of areas of care that could be improved.**

- Appropriate escalation of early warning scores.
- Time to consultant review.
- Communication between clinical teams.
- End-of-life discussions with patients and their families.
- DNACPR decision-making.
- Use of, and compliance with, care bundles.
- Timely access to CT scan.
- Fluid balance monitoring.
- Delays in assessment and initial treatment.
- Timely administration of antibiotics.
- Communication between clinical teams.
- Weekend medical cover and access to theatres.
- Advance care planning for patients in care homes.

**Examples of other areas that impact on care.**

- Quality of documentation.
- Recording of comorbidities.
- Coding accuracy.

Comparing sepsis alerts with all other alerts investigated, a higher proportion of trusts (85%, 16/19) (table 3) reported deficiencies in care (p=0.122) (the difference was not statistically significant). A lower proportion of trusts (52%, 14/27) with multiple (combined) alerts reported deficiencies in care compared with 75% for a single alert (91/122) (p=0.019).

**Table 3:** Care Quality Commission findings of alerts sent 2011–2013 for all diagnosis/procedure groups, single, combined multiple* and sepsis

	All alerts	Multiple index alerts*	Sepsis†	
	n=154	n=32	n=19	
	Total	Single	Combined multiple*	Sepsis†
Care	51 (33%)	42 (34%)	9 (33%)	10 (53%)
Care and coding	55 (36%)	49 (40%)	5 (19%)	6 (32%)
Coding only	22 (14%)	18 (15%)	4 (15%)	1 (5%)
Casemix	3 (2%)	1 (1%)	2 (7%)	0 (0%)
No review	3 (2%)	0 (0%)	2 (7%)	0 (0%)
No improvement required	17 (11%)	11 (9%)	4 (15%)	1 (5%)
No cause stated	3 (2%)	1 (1%)	1 (4%)	1 (5%)
Total	154	122	27	19

*Multiple alerts are two or more alerts generated within a year of each other for a trust for the same condition. These alerts were combined and treated as one event.

†Septicaemia (except in labour).

Coding issues were identified in 50% (77/154) of cases. A lower proportion of trusts with multiple (combined) alerts reported coding issues (33%, 9/27) compared with 55% with single alerts (67/122) (p=0.042).

Twenty (13%) cases reported that no areas for improvement were identified or where casemix only was deemed the issue. The proportion of trusts reporting ‘casemix’ or ‘no improvement required’ was higher in multiple (combined) alerts (22%, 6/27) compared with single alerts (10%, 12/122) (p=0.074). According to trust reports, deficiencies in care may have been related to patient outcomes in 25% (38/154) of the investigated alerts.

Action plans acceptable to the CQC were created in 64% (98/154) of the cases after the initial investigation and 77% (118/154) by the time the cases were closed. Where care was identified as an issue, 85% of cases (90/106) had action plans in place, acceptable to the CQC by the end of the study period. In 13 of the remaining cases, although plans were present, the CQC determined that they were incomplete or lacked time scales for action. Action plans were created in 58% (28/48) of those cases where areas of care were not cited as needing improvement. In 17 of these cases, coding was cited as a key factor for the outcome, while for the remaining 11 cases the findings included ‘no improvement required’ and casemix.

## Discussion

### Key findings

From the inception of the Imperial College Mortality Surveillance System in October 2016, 860 mortality alerts have been generated, of which 76% were sent to trusts. The majority of acute NHS trusts in England (134/154) have received an alert since the alerting system began, and 43 (30%) trusts received more than one alert for a single condition within 12 months. In a subset of 204 alerts sent to trusts between 2011 and 2013, 154 (75%) were investigated by the CQC. Of these pursued alerts, hospital trusts’ local investigations found areas of care that could be improved in 69% of all alerts, 84% of sepsis alerts and 52% of multiple alerts. Care was considered, by the trusts themselves, to potentially have been related to patient outcomes in 25% of all investigated alerts. The CQC investigations resulted in action plans in 77% of all pursued cases.

### Findings in relation to other studies

Our study systematically reviewed evidence held within a national independent health regulator (CQC), covering communications with hospitals relating to a mortality surveillance system, resulting in full data capture over a study period. By reviewing the documents held by the CQC, we have built up a clearer understanding of the complexities of the mortality surveillance system as an intervention. We also gained an insight into some of the consequences of our alerts. For example, trusts were usually able to identify areas for improvement within their care pathways and implemented action plans to address these issues. Some trusts have independently reported details of their responses to mortality alerts, which support our findings. For example, Northern Devon Healthcare published a mortality report in response to the CQC after an Imperial College Mortality Alert in 2015.^14^ It investigated all deaths that were identified in the mortality alert letter. Each death was scored against the Hogan Scale, National Confidential Enquiry into Patient Outcome and Death (NCEPOD) and Hogan Quality Scale (as requested by the CQC). The audit did not identify any ‘preventable’ deaths, but there was evidence that the quality of care could have been better. The audit also flagged up issues with coding.

### Interpretation of findings

This is the first comprehensive analysis of a national mortality surveillance system for hospitals. This descriptive study was not enough to determine whether the surveillance system is effective at identifying poor care. An alternative approach, such as random case note audits in alerting compared with non-alerting hospitals, might provide stronger evidence of whether the surveillance system identifies care problems any more effectively than other approaches. Structured case note reviews of hospital deaths have been used to identify cases judged to be preventable with ordinary standards of care,^15^ and found that death was potentially avoidable in around 5% of all inpatient deaths.^16^ Although Hogan *et al* 16 found little evidence that these ‘avoidable’ deaths were associated with measures of hospital quality, this could be due to their selection of quality indicators, the very narrow definition of preventable death or the lack of statistical power in the study. Interestingly the researchers did find evidence of avoidable deaths in 100% of hospitals sampled, which may reflect their more systematic approach to case note reviews. A combination of a structured case note review and statistical alerts may therefore be a helpful tool to systematically identify quality of care problems, particularly for assessing the quality of care for conditions with high case fatality.^17^

We found that most trusts (87%) had received an alert letter at some point in time between 2007 and 2016. However, the fact that a trust did not receive an alert letter is no guarantee that a trust is without quality issues. The threshold for an alert is very high; we base our threshold on a false alarm rate of 0.1%, which limits the system’s sensitivity, but decreases the risk of a false alert.

### Strengths and weaknesses

In our review of the CQC communications with trusts, decisions on the classifications of findings were carried out by a single researcher, but our methodological approach to categorising subjective findings will limit potential biases. The local trust investigation process will, however, be prone to bias. An example of this might be hindsight bias. The fact that an alert has been triggered will mean that hospital case note reviews may be looking for potential issues. In the absence of controls, we cannot ascertain whether these issues also occur in non-alerting trusts.

Given that Imperial College produces the alerts, there is potential for bias in the interpretation of any evaluation of the system by Imperial researchers. To mitigate this potential bias, the research team included two independent researchers from external institutions (AE and CV) and an independent Scientific Advisory Committee to oversee and advise on the conduct of the research.

Our study found that in 25% of the pursued alerts, trusts reported that they considered that deficiencies in care may have been related to patient outcomes. The remaining 75% of trusts did not report such a relationship. Although we cannot be certain, it seems likely that these trusts did not consider that quality of care contributed to their outcomes.

Our study found that action plans were created in 64% of CQC cases after the initial investigation but 77% by the time the cases were closed. This suggests that the CQC may be more likely to pursue the case until an acceptable action plan is created. We are unable to determine whether it was our alerts alone or CQC’s pursuance of alerts that triggered the creation of action plans. We also found action plans were created in 58% of cases (28/48) where areas of care were not cited as needing improvement. In the majority of these cases (17/28), coding was cited as a factor and action plans may have focused on improving coding. However, there were still some cases (11/28) where the case note reviews concluded that outcomes were due to the patient casemix, or overall there was no improvement required but action plans were still created. These could be due to the CQC being notified of actions plans which do not directly relate to the case under investigation, for example action plans already under way or as a result of identified opportunities to make improvements to a service.

The creation of action plans to improve care in ‘outlier’ trusts is highlighted in the CQC annual reports.^11 18^ We noted from this that there was huge variation in the case note review standards used by the trusts in their responses. For example, some trusts reported they considered the findings from their audit may have been related to patient outcomes, whereas others did not consider this.

### Implications for clinicians and policymakers

Over the last 9 years most trusts (87%) have experienced the process of receiving a mortality alert. This work enables trust staff to more fully understand the meaning of an alert, the process for communicating with the CQC and the potential implications for care. We have shown the interacting factors within the mortality surveillance system and how the CQC follows up on alerts, including the length of time taken for the process to be completed.

For policymakers this descriptive analysis highlights the resources used as part of Imperial College Mortality Surveillance System, and will be helpful when assessing this and future systems for monitoring patient safety. Overall, our research finds that alerts followed up by the CQC often result in trusts identifying problems in care (69%), indicating value in the system.

### Unanswered questions and future research

We hypothesised that if multiple alerts are indicating a persistent problem within a hospital, the CQC findings would indicate an issue either with quality of care or coding. However, we found that hospitals were less likely to report care or coding issues if they alerted more than once for the same condition within 12 months. The number of multiple alerts was small and the associations were borderline. However, if true, further research is required to understand why trusts that receive multiple alerts are less likely to unearth quality issues. We cannot yet be certain whether the mortality alerts are highlighting areas of substandard care or whether the process of completing case note reviews in response to the CQC requests is simply to highlight areas in care that could be improved regardless of whether these problems were implicated in the mortality alerts. Only an independent comparison of case notes in trusts that alert and trusts not alerting in specific diagnosis or procedure groups could answer this question definitively.

Our study did not establish whether action plans were implemented. Previous research has questioned the value of root cause analysis.^19^ Our paper, however, does not set out to discuss the method of investigation used by hospitals, to document the changes in patient care within the alerting hospital trusts or to measure changes in outcome. The study is part of a larger project,^20^ which does explore alert response and outcomes. Elsewhere in our project, the response to a mortality alert was considered using indepth qualitative interviews with key stakeholders within trusts.^20^ We undertook 11 institutional case studies in trusts that alerted for two target conditions: sepsis and acute myocardial infarction (AMI). The alerts triggered institutional responses across all the case study sites. These responses included infrastructure changes; changes in patient pathways; changes in diagnosis of sepsis and AMI; and improvement to and training in case note review and coding. Also investigated were the subsequent trends in mortality after alert notification using interrupted time analysis and the relation between mortality alerts and other measures of quality.^21^

## Conclusion

The mortality surveillance system has generated a large number of alerts since 2007. The most common alert was for sepsis, and most trusts have received at least one mortality alert since the system began. The letter from Imperial College London that accompanies each alert suggests a number of possible explanations for the findings, including random variation, poor data quality or coding problems, and casemix issues. This study finds that following receipt of an alert letter, local trust investigations find areas of care that could be improved in nearly 70% of these investigations. Identifying whether mortality alerts are the most efficient means to highlight areas of substandard care will require further investigation.
