# Supplementary material for: National hospital mortality surveillance system: a descriptive analysis
Source: BMJ Qual Saf. 2018 Oct 8;27(12):974–81. doi: 10.1136/bmjqs-2018-008364 (PMC6288692; doi:10.1136/bmjqs-2018-008364)
Supplement: Supplementary data [file bmjqs-2018-008364supp002.pdf]

Supplementary file 1

List of diagnoses and procedures in Mortality Alerting System

Group No	Description
**Diagnoses**	
**2**	Septicaemia (except in labour)
**49**	Diabetes mellitus without complication
**50**	Diabetes mellitus with complications
**55**	Fluid and electrolyte disorders
**59**	Deficiency and other anaemia
**66**	Alcohol-related mental disorders
**67**	Substance-related mental disorders
**69**	Affective disorders
**70**	Schizophrenia and related disorders
**71**	Other psychoses
**72**	Anxiety, somatoform, dissociative, and personality disorders
**83**	Epilepsy, convulsions
**96**	Heart valve disorders
**100**	Acute myocardial infarction
**101**	Coronary atherosclerosis and other heart disease
**103**	Pulmonary heart disease
**106**	Cardiac dysrhythmias
**109**	Acute cerebrovascular disease
**114**	Peripheral and visceral atherosclerosis
**115**	Aortic, peripheral, and visceral artery aneurysms
**125**	Acute bronchitis
**127**	Chronic obstructive pulmonary disease and bronchiectasis
**128**	Asthma
**130**	Pleurisy, pneumothorax, pulmonary collapse
**144**	Regional enteritis and ulcerative colitis
**145**	Intestinal obstruction without hernia

**148**	Peritonitis and intestinal abscess
**149**	Biliary tract disease
**150**	Liver disease, alcohol-related
**153**	Gastrointestinal haemorrhage
**154**	Non-infectious gastroenteritis
**157**	Acute and unspecified renal failure
**158**	Chronic renal failure
**159**	Urinary tract infections
**196**	Normal pregnancy and/or delivery
**197**	Skin and subcutaneous tissue infections
**199**	Chronic ulcer of skin
**205**	Spondylosis, intervertebral disc disorders, other back problems
**218**	Liveborn
**226**	Fracture of neck of femur (hip)
**233**	Intracranial injury
**237**	Complication of device, implant or graft
**238**	Complications of surgical procedures or medical care
**Procedure**	
**2**	Operations on peptic ulcer
**3**	Excision of stomach (non-bariatric)
**4**	Therapeutic operations on jejunum and ileum
**5**	Appendicectomy
**8**	Laparascopic cholecystectomy
**9**	Total excision of spleen
**10**	Transplantation of kidney
**11**	Placement of ambulatory apparatus for compensation for renal failure
**12**	Graft of bone marrow
**13**	Amputation of leg
**14**	Excision of lung
**15**	Plastic repair of mitral valve (adult without CABG)
**16**	Plastic repair of aortic valve (adult without CABG)
**19**	Excision of oesophagus +/- stomach
**20**	Excision of larynx or pharynx

**21**	Excision of tongue
**23**	Excision of pancreas or head of pancreas
**24**	Partial excision of liver
**25**	Total excision of kidney
**26**	Abdominal excision of uterus
**27**	Total excision of bladder
**28**	Hip replacement
**29**	Knee replacement
**30**	Iliac bypass (without transluminals)
**31**	Coronary angioplasty (PTCA)
**35**	Reconstruction of carotid artery
**39**	Craniotomy for trauma
**40**	Clip and coil aneurysms
**47**	CABG (isolated first time)
**201**	Extirpation of lesion of meninges of brain
**202**	Shunting for hydrocephalus
**203**	Primary neck procedures
**507**	Transurethral resection of bladder tumour (TURBT)
**601**	Destruction of lesion of retina
**602**	Extraction of tooth
**606**	Cardiac pacemaker or defibrillator introduced through the vein
**608**	Vaginal excision of uterus (without repair of prolapse)
**610**	Therapeutic endoscopic procedures on upper GI tract
**613**	Therapeutic endoscopic procedures on lower GI tract
**614**	Repair of umbilical hernia
**616**	Therapeutic endoscopic procedures on biliary tract
**620**	Primary repair of tendon
**622**	Reduction of fracture of bone (upper/lower limb)
**623**	Excision of breast
**624**	Therapeutic endoscopic procedures on ureter
**626**	Endoscopic resection of outlet of male bladder
**629**	Excision of cervix uteri
**630**	Vaginal prolapse repair +/- hysterectomy

**632**	Termination of pregnancy (other)
**633**	Delivery
**634**	Head of femur replacement
**646**	Transluminal operations on femoral artery
**649**	Paediatric open heart operations (excl. transplants)
**651**	Lumbar spine procedures
**671**	Transplantation of lung
**672**	Transplantation of liver
**674**	Transplantation of heart +/- lung
**677**	Repair of abdominal aortic aneurysm (AAA)
**680**	Electroconvulsive therapy
**699**	Repair of thoracic or unspecified aortic aneurysm
**700**	Infra-inguinal bypass
**701**	Other femoral bypass
**702**	Correction of deformity of eyelid or ptosis
**703**	Other destruction of haemorrhoid
**705**	Primary repair of femoral hernia
**707**	Excision of thyroid gland
**708**	Repair of other hernia of abdominal wall
**709**	Other excision of gall bladder
**710**	Excision of colon and/or rectum
**711**	CABG (other)
**714**	Excision of ovary and/or fallopian tube (without hysterectomy)
**716**	Percutaneous transluminal ablation of heart
**728**	Therapeutic endoscopic operations on urethra
**731**	Vaginal operations to support outlet of female bladder
**739**	Primary repair of incisional hernia
**751**	Reduction of fracture of neck of femur
**752**	Therapeutic transluminal operations on iliac artery
**753**	Therapeutic endoscopic operations on larynx
**755**	Bariatric operations

NB. Not all conditions/procedures have triggered a Mortality Alert since monitoring began in 2007
